# Initial Evaluation of [^18^F]FAPI-74 PET for Various Histopathologically Confirmed Cancers and Benign Lesions

**DOI:** 10.2967/jnumed.123.265486

**Published:** 2023-08

**Authors:** Tadashi Watabe, Sadahiro Naka, Mitsuaki Tatsumi, Takashi Kamiya, Toru Kimura, Yasushi Shintani, Kaori Abe, Tomohiro Miyake, Kenzo Shimazu, Shogo Kobayashi, Yukinori Kurokawa, Hidetoshi Eguchi, Yuichiro Doki, Hidenori Inohara, Hiroki Kato, Yuriko Mori, Jens Cardinale, Frederik L. Giesel

**Affiliations:** 1 Department of Nuclear Medicine and Tracer Kinetics, Graduate School of Medicine, Osaka University, Osaka, Japan;; 2 Institute for Radiation Sciences, Osaka University, Osaka, Japan;; 3 Department of Pharmacy, Osaka University Hospital, Osaka, Japan;; 4 Department of Radiology, Osaka University Hospital, Osaka, Japan;; 5 Department of Thoracic Surgery, Graduate School of Medicine, Osaka University, Osaka, Japan;; 6 Department of Breast and Endocrine Surgery, Graduate School of Medicine, Osaka University, Osaka, Japan;; 7 Department of Gastroenterological Surgery, Graduate School of Medicine, Osaka University, Osaka, Japan;; 8 Department of Otorhinolaryngology–Head and Neck Surgery, Graduate School of Medicine, Osaka University, Osaka, Japan; and; 9 Department of Nuclear Medicine, University Hospital Duesseldorf, Heinrich Heine University, Duesseldorf, Germany

**Keywords:** fibroblast activation protein, [^18^F]FAPI-74, cancer-associated fibroblast, PET, oncology

## Abstract

The ^18^F-labeled fibroblast activation protein inhibitor (FAPI) [^18^F]FAPI-74 has the benefit of a higher synthetic yield and better image resolution than ^68^Ga-labeled FAPI. We preliminarily evaluated the diagnostic performance of [^18^F]FAPI-74 PET in patients with various histopathologically confirmed cancers or suspected malignancies. **Methods:** We enrolled 31 patients (17 men and 14 women) with lung cancer (*n* = 7), breast cancer (*n* = 5), gastric cancer (*n* = 5), pancreatic cancer (*n* = 3), other cancers (*n* = 5), and benign tumors (*n* = 6). Twenty-seven of the 31 patients were treatment-naïve or preoperative, whereas recurrence was suspected in the remaining 4 patients. Histopathologic confirmation was obtained for the primary lesions of 29 of the 31 patients. In the remaining 2 patients, the final diagnosis was based on the clinical course. [^18^F]FAPI-74 PET scanning was performed 60 min after the intravenous injection of [^18^F]FAPI-74 (240 ± 31 MBq). The [^18^F]FAPI-74 PET images were compared between the primary or local recurrent lesions of malignant tumors (*n* = 21) and nonmalignant lesions (*n* = 8: type-B1 thymomas, granuloma, solitary fibrous tumor, and postoperative or posttherapeutic changes). The uptake and number of detected lesions on [^18^F]FAPI-74 PET were also compared with those on [^18^F]FDG PET for available patients (*n* = 19). **Results:** [^18^F]FAPI-74 PET showed higher uptake in primary lesions of various cancers than in nonmalignant lesions (median SUV_max_, 9.39 [range, 1.83–25.28] vs. 3.49 [range, 2.21–15.58]; *P* = 0.053), but some of the nonmalignant lesions showed high uptake. [^18^F]FAPI-74 PET also showed significantly higher uptake than [^18^F]FDG PET (median SUV_max_, 9.44 [range, 2.50–25.28] vs. 5.45 [range, 1.22–15.06] in primary lesions [*P* = 0.010], 8.86 [range, 3.51–23.33] vs. 3.84 [range, 1.01–9.75] in lymph node metastases [*P* = 0.002], and 6.39 [range, 0.55–12.78] vs. 1.88 [range, 0.73–8.35] in other metastases [*P* = 0.046], respectively). In 6 patients, [^18^F]FAPI-74 PET detected more metastatic lesions than [^18^F]FDG PET. **Conclusion:** [^18^F]FAPI-74 PET showed higher uptake and detection rates in primary and metastatic lesions than did [^18^F]FDG PET. [^18^F]FAPI-74 PET is a promising novel diagnostic modality for various tumors, especially for precise staging before treatment, including characterization of tumor lesions before surgery. Moreover, ^18^F-labeled FAPI ligand might serve a higher demand in clinical care in the future.

## Visual Abstract

**Figure:**
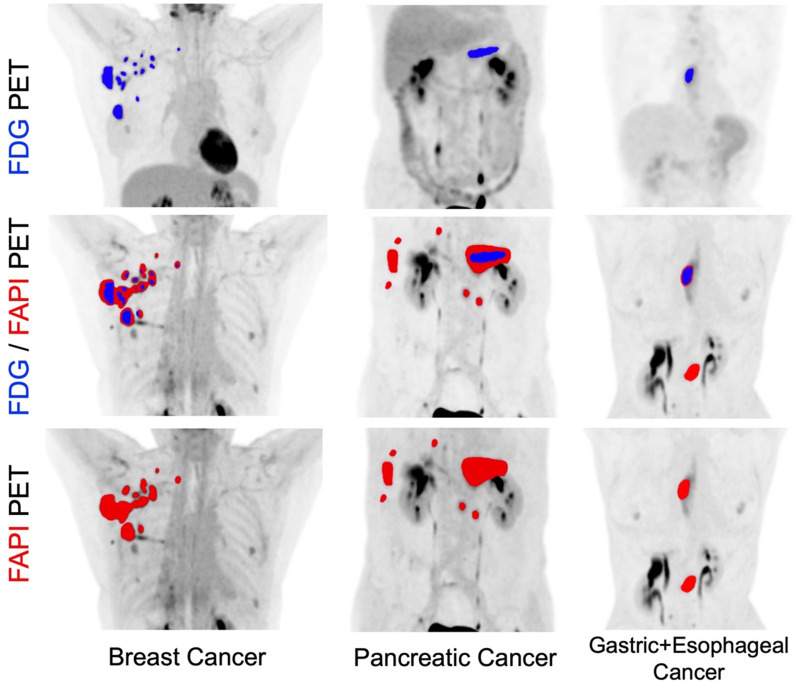

Cancer-associated fibroblasts are major components of the cancer stroma and play an important role in cancer invasion and metastasis in the tumor microenvironment (1). Cancer-associated fibroblasts interact with cancer cells by secreting numerous chemokines and cytokines, such as transforming growth factor β, inducing immunosuppression in the tumor microenvironment (1). Cancer-associated fibroblasts express fibroblast activation protein (FAP), and the expression levels of FAP have been reported to correlate with the prognosis in patients with cancer (1). In addition, FAP expression has been confirmed in various cancer types, with minimal expression observed in normal organs (2*,*^3^). The FAP inhibitor (FAPI) has gained attention as an excellent PET probe that can accurately detect many types of cancer compared with the conventional glucose analog [^18^F]FDG. ^68^Ga-labeled FAPI ([^68^Ga]FAPI-04 or [^68^Ga]FAPI-46) and ^18^F-labeled FAPI ([^18^F]FAPI-74) are most commonly available for clinical use (4–6). Recently, the number of published papers reporting the excellent performance of FAPI PET has increased, and clinical trials are being conducted (NCT05262855 and NCT05641896). FAPI PET is expected to be used increasingly in cancer diagnosis and treatment planning for optimized patient management.

However, the short half-life of ^68^Ga (68 min) can cause problems in production and delivery. It requires onsite production by a ^68^Ga generator or cyclotron production using a solid target of ^68^Zn. It also has a limitation in that one production cycle of ^68^Ga allows the acquisition of only 2–3 patient scans because of the relatively low yield and short half-life. Labeling with ^18^F (half-life, 110 min), such as [^18^F]FDG, will enable more practical large-scale production. For prostate-specific membrane antigen PET, [^68^Ga]PSMA-11 is being replaced by ^18^F-labeled prostate-specific membrane antigen probes, such as [^18^F]DCFPyL, because of the increasing cost of ^68^Ga generators. Thus, ^18^F-labeled FAPI ligands, such as [^18^F]FAPI-74, may be the best option for widespread use in the global market with the added benefit of a higher synthetic yield and delivery from centralized large-scale production (5).

In this study, we preliminarily evaluated the diagnostic performance of [^18^F]FAPI-74 PET in patients with various histopathologically confirmed cancers or suspected malignancies.

## MATERIALS AND METHODS

### Patients

We enrolled 31 patients (age range, 38–83 y; 17 men and 14 women) in this prospective study. The inclusion criteria were as follows: patients who had been diagnosed with a malignant tumor or suspected malignancy before treatment and had undergone CT or [^18^F]FDG PET, patients who had been diagnosed with a malignant tumor and had undergone or were to undergo chemotherapy or radiotherapy, and patients with suspected recurrence based on the clinical findings or other diagnostic imaging, such as CT or [^18^F]FDG PET, after treatment. The patient characteristics are summarized in Table 1. The study included patients with lung cancer (*n* = 7), breast cancer (*n* = 5), gastric cancer (*n* = 5), pancreatic cancer (*n* = 3), other cancers (*n* = 5), and benign tumors (*n* = 6). Twenty-seven of the 31 patients were treatment-naïve or preoperative, whereas recurrence was suspected in the remaining 4 patients. Patients who were pregnant or suspected to be pregnant, pediatric patients who required sedation, and patients considered unsuitable for participation in the study were excluded. Histopathologic confirmation was obtained for the primary lesions of 29 of the 31 patients (94%) and for the metastatic lesions of 10 patients who underwent surgical resection. In 2 patients (2/31), the final diagnosis was based on the clinical course, including the results of follow-up imaging. Postoperative fibrosis was suspected in 1 patient because no remarkable changes were observed on the follow-up MRI. In another case, the patient had been followed up for more than 10 y after surgery for breast cancer. Multiple metastases in the lymph nodes (LNs), liver, and bone were detected on CT and [^18^F]FDG PET in this patient. Among the patients with LN metastasis (*n* = 12), the histopathology of the LN was confirmed via surgical resection or biopsy in 6 patients and was based on comprehensive interpretation by a nuclear medicine specialist who reported a high possibility of metastasis in the other 6 patients.

**TABLE 1.** Patient Characteristics

Characteristic	Data
Number of patients	31
Sex	
Male	17
Female	14
Age range (y)	38–83 (median, 68)
Diagnosis	Lung cancer (adenocarcinoma [*n* = 6] and squamous cell carcinoma [*n* = 1]), breast cancer (invasive ductal carcinoma [*n* = 5]), gastric cancer (*n* = 5), pancreatic cancer (adenocarcinoma [*n* = 3]), oropharyngeal cancer (*n* = 1), thyroid cancer (papillary carcinoma [*n* = 1]), thymic cancer and thymoma (type B3) (*n* = 3), and benign tumors (*n* = 6)
Patient status	Before treatment or surgery (*n* = 27, including after chemotherapy [*n* = 5] or after chemoradiation therapy [*n* = 1]); suspected recurrence (*n* = 4)
Histologic findings of benign tumors	Thymoma (type B1 [*n* = 2]), granuloma (*n* = 1), solitary fibrous tumor (*n* = 1), and postoperative changes (*n* = 2)

The study protocol was approved by the Institutional Review Board of the Osaka University Hospital (approval 21472-4), and the study was performed in accordance with the ethical standards laid down in the 1964 Declaration of Helsinki. Written informed consent was obtained from all patients before their inclusion in the study.

### Synthesis of [^18^F]FAPI-74 and [^18^F]FDG

[^18^F]FAPI-74 solution was synthesized using CFN-MPS200 (Sumitomo Heavy Industries), according to a previous study (6). [^18^F]fluoride eluted with 0.5 M sodium acetate buffer and precursor solution (a mixture of dimethyl sulfoxide, 10 mM aluminum chloride, 20% w/v ascorbic acid, and 4 mM FAPI-74 precursor) was mixed and fluorinated for 5 min at room temperature, followed by 15 min at 95°C. [^18^F]FAPI-74 was purified from the reaction solution using an hydrophilic–lipophilic balance cartridge and ethanol. Finally, the [^18^F]FAPI-74 solution was obtained by diluting with 10 mM phosphate-buffered saline (pH 6.7) containing 100 mg of sodium ascorbate and filtering through a sterile 0.22-μm Millex GV filter. The radioactivity of the [^18^F]FAPI-74 solution was 10,201 ± 593 MBq at the end of synthesis by the irradiation condition at 25 μA for 30–35 min. The radiochemical purity was 97.3% ± 0.3%.

The [^18^F]FDG solution was synthesized using the F200 synthesizer (Sumitomo Heavy Industries). After eluting [^18^F]fluoride with Kryptofix 222 (Merck KGaA) and potassium carbonate solution and drying completely, we fluorinated [^18^F]FDG with mannose triflate and then deprotected with 0.3 M NaOH. [^18^F]FDG was subsequently purified using solid-phase extraction cartridges (IC-H, PS-2, and Alumina N) and passed through a sterile 0.22-μm vented Millex GS filter. The radiochemical purity of [^18^F]FDG was more than 95%.

### PET/CT Scanning

PET/CT scanning was performed 60 min after the intravenous injection of [^18^F]FAPI-74 (240 ± 31 MBq). The patients were monitored for adverse events from the time of administration to the end of the PET scan. PET/CT images were acquired using a Biograph Vision 600 (Siemens Healthineers) in continuous bed motion (matrix, 440 × 440; pixel size, 1.65 mm) at 3.5 mm/s for 4 frames. The PET images were reconstructed to a slice thickness of 3 mm with an increment of 3 mm using ordered-subset expectation maximization with 3 iterations per 5 subsets and was gauss-filtered to a transaxial resolution of 3 mm in full width at half maximum. Attenuation was corrected using unenhanced low-dose CT data (effective dose, 50 mAs). The CT scans were reconstructed to a slice thickness of 3 mm in increments of 3 mm. [^18^F]FDG PET scanning was performed 60 min after the intravenous injection of [^18^F]FDG (3.7 MBq/kg) according to the institutional protocol.

### Image Analysis

SUV measurements were performed via volume-of-interest analysis of the [^18^F]FAPI-74 PET images using Syngovia software (Siemens Healthineers). SUV_max_ and the tumor–to–normal background (T/N) ratio were compared between the primary or local recurrent lesions of malignant tumors (*n* = 21) and nonmalignant lesions (*n* = 8). Volumes of interest of normal background were placed on the normal areas surrounding each tumor lesion where physiologic accumulation did not overlap. We defined nonmalignant lesions as postoperative/therapeutic changes and benign/low-malignancy tumors, such as thymoma (type B1), granuloma, and solitary fibrous tumor (intermediate lesion with low malignancy potential). The SUV_max_ of the primary lesions, LN metastases, and other metastases, as well as the number of detected metastatic lesions on [^18^F]FAPI-74 PET, were compared with those of [^18^F]FDG PET (within 8 wk) for available cases of malignant tumors (*n* = 19). The median interval between [^18^F]FAPI-74 PET and [^18^F]FDG PET was 18 d (range, 6 − 56 d). [^18^F]FDG PET images obtained using different PET scanners (*n* = 12) were also included.

### Statistical Analyses

Comparisons between the 2 groups were performed using the Wilcoxon signed-rank test for paired data or Mann–Whitney *U* test for unpaired data by SPSS (version 25.0; IBM Corp.). The differences were considered statistically significant at a *P* value of less than 0.05.

## RESULTS

All patients were monitored for adverse or serious adverse events due to the [^18^F]FAPI-74 injection. The symptoms were monitored after injection until the end of the PET scan, and no adverse events occurred due to its administration. No clinically significant changes were observed in heart rate, oxygen saturation, or body temperature before or after administration of the [^18^F]FAPI-74 solution (Supplemental Table 1; supplemental materials are available at http://jnm.snmjournals.org).

**FIGURE 1.**
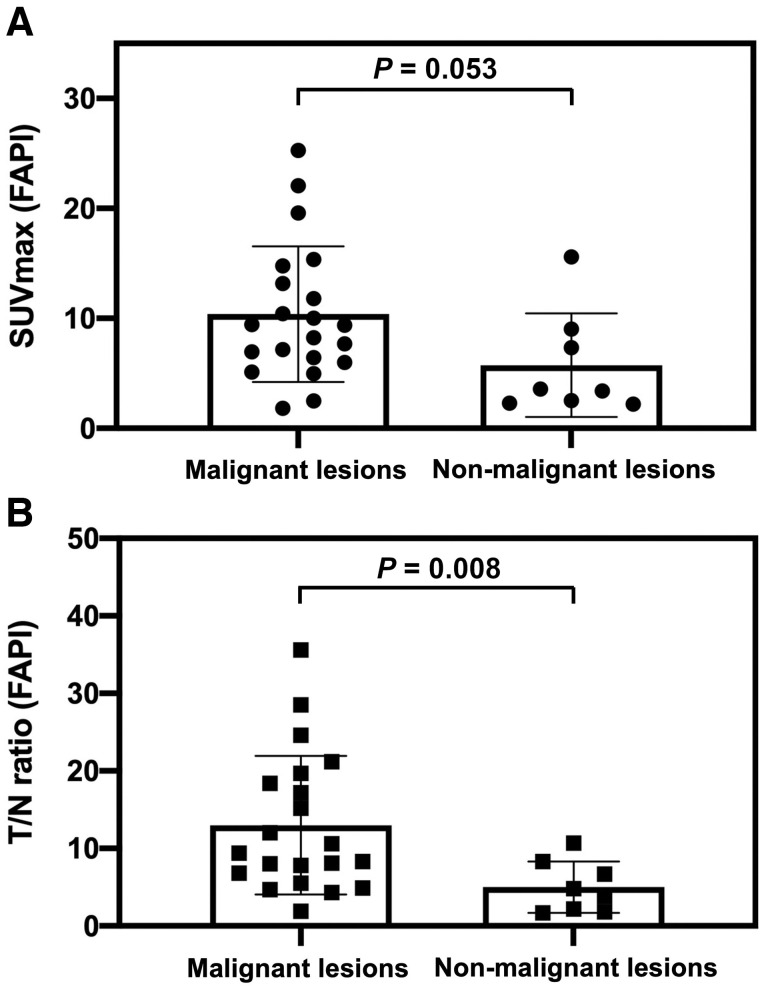
Comparison of uptake on [^18^F]FAPI-74 PET between malignant tumors and nonmalignant lesions: SUV_max_ (A) and T/N ratio (B) (with *P* value by Mann–Whitney *U* test).

**FIGURE 2.**
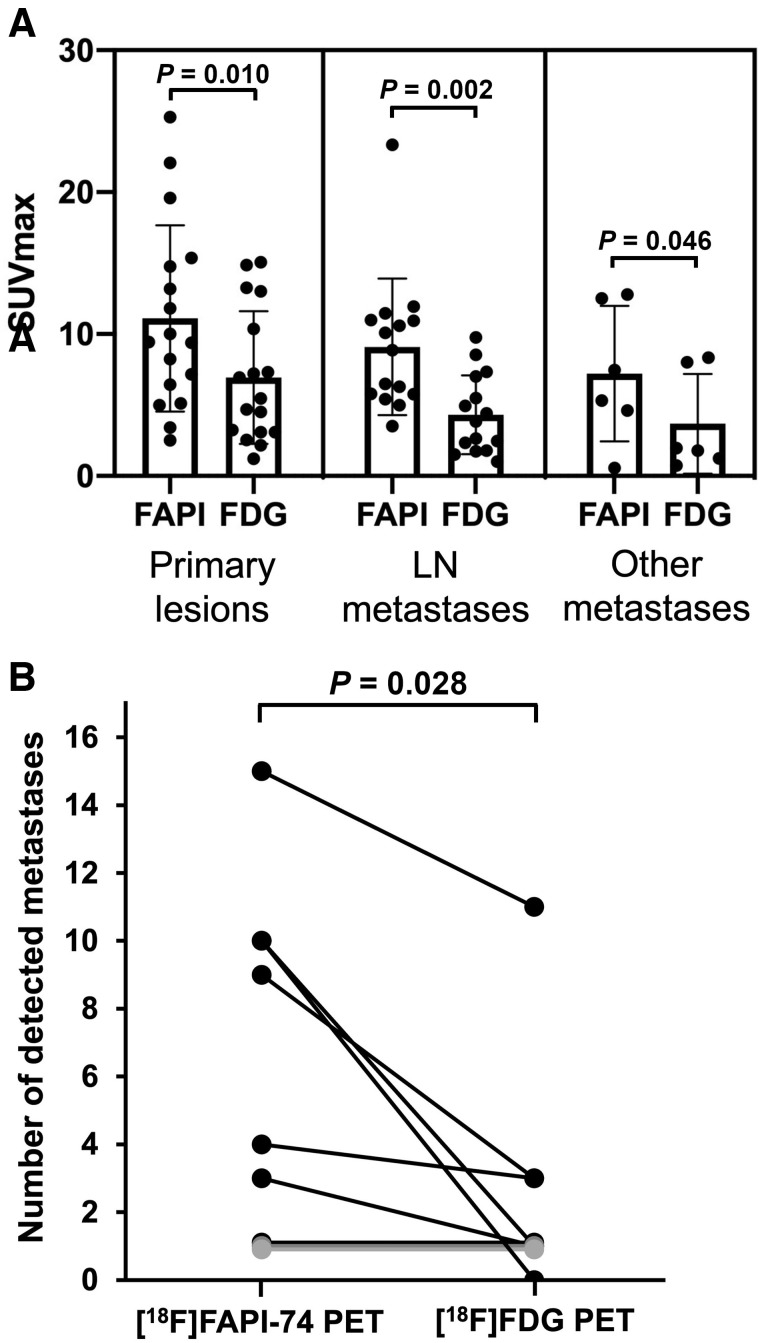
Comparison of uptake between [^18^F]FAPI-74 PET and [^18^F]FDG PET: SUV_max_ in primary lesions, LN metastases, and other metastases (A) and number of detected metastatic lesions on [^18^F]FAPI-74 PET compared with [^18^F]FDG PET (B) (with *P* value by Wilcoxon signed-rank test).

**FIGURE 3.**
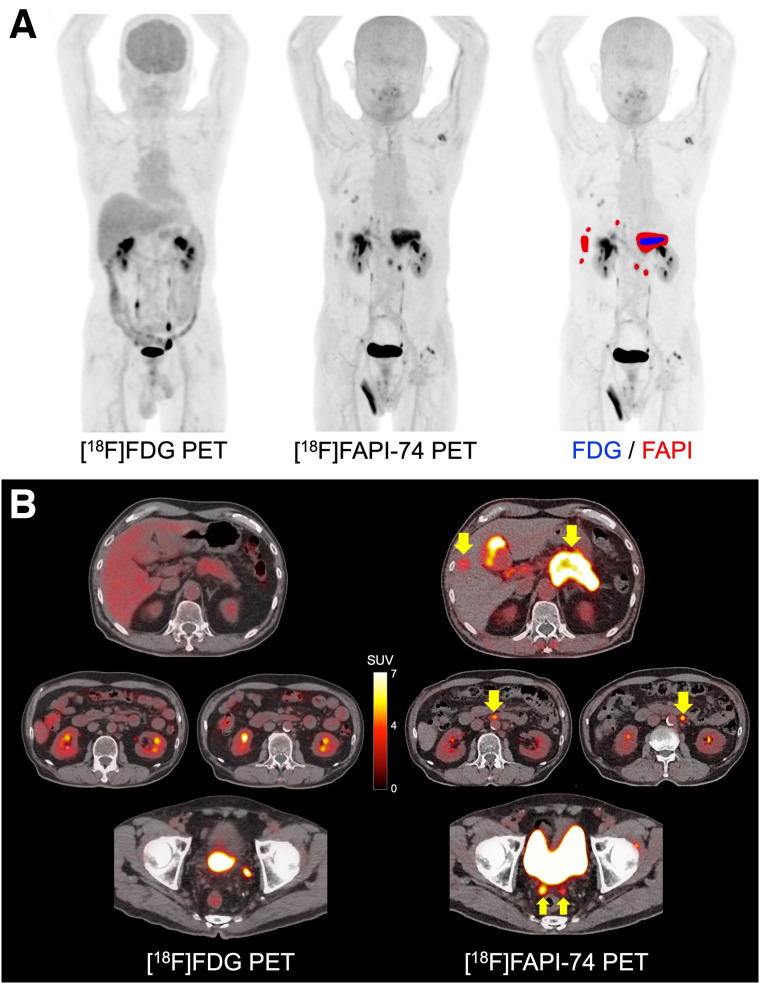
A 68-y-old man with multiple LN and peritoneal metastases of pancreatic cancer. (A) Comparison of maximum-intensity projection images: image on right shows fusion with positive lesions on [^18^F]FAPI-74 PET (red area) and [^18^F]FDG PET (blue area). (B) PET/CT images on [^18^F]FAPI-74 PET and [^18^F]FDG PET (arrows indicate metastatic lesions). [^18^F]FAPI-74 PET detected more metastatic lesions than [^18^F]FDG PET (SUV_max_ of primary lesion is 9.4 and 3.2, respectively). Brain uptake is decreased on [^18^F]FDG PET because of high blood glucose level (277 mg/dL).

**FIGURE 4.**
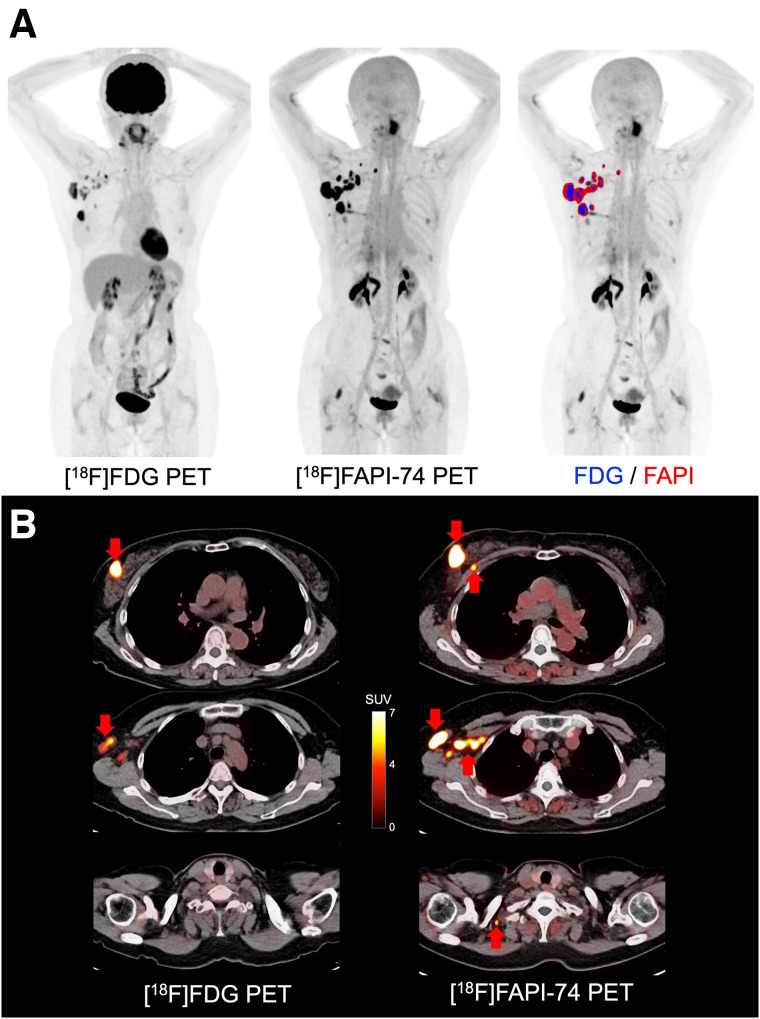
A 59-y-old woman with multiple LN metastases of breast cancer. (A) Comparison of maximum-intensity projection images: image on right shows fusion with positive lesions on [^18^F]FAPI-74 PET (red area) and [^18^F]FDG PET (blue area), (B) PET/CT images on [^18^F]FAPI-74 PET and [^18^F]FDG PET (arrows indicate metastatic lesions). [^18^F]FAPI-74 PET detected more metastatic lesions than [^18^F]FDG PET (SUV_max_ of primary lesion is 19.6 and 13.0, respectively).

**FIGURE 5.**
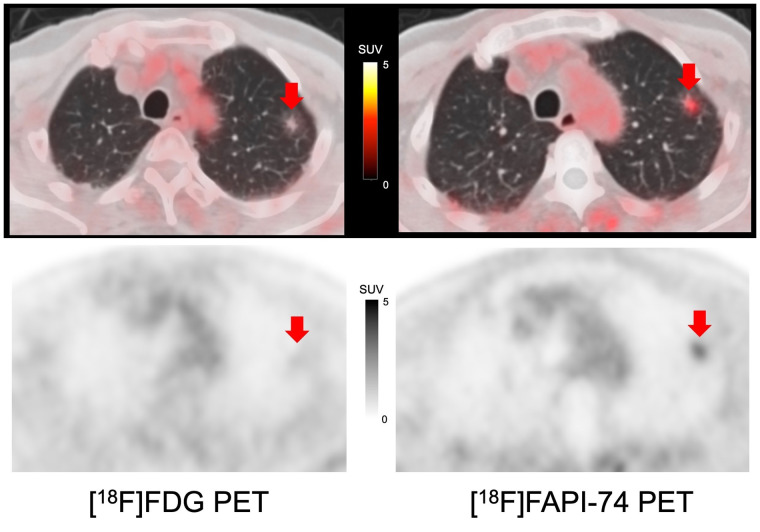
Comparison of PET/CT images on [^18^F]FAPI-74 PET and [^18^F]FDG PET of 80-y-old man with lung adenocarcinoma with ground-glass opacity (arrows indicate primary lesions). [^18^F]FAPI-74 PET showed clearer uptake than [^18^F]FDG PET (SUV_max_ of primary lesion is 2.5 and 1.2, respectively).

In the comparison between malignant tumors and nonmalignant lesions, [^18^F]FAPI-74 PET showed higher uptake in primary lesions of various cancers (median SUV_max_, 9.39 [range, 1.83–25.28]; median T/N ratio, 9.36 [range, 1.87–35.61]) than that in nonmalignant lesions (median SUV_max_, 3.49 [range, 2.21–15.58]; T/N ratio, 4.27 [range, 1.67–10.74]) (*P* = 0.053 and *P* = 0.008, respectively) (Figs. 1A and B). However, some of the benign tumors and treatment-related changes showed high uptake on [^18^F]FAPI-74 PET, such as granuloma, postoperative changes, and radiation pneumonitis (Supplemental Table 2). Compared with [^18^F]FDG PET, [^18^F]FAPI-74 PET also showed significantly higher uptake in the primary lesions (median SUV_max_, 9.44 [range, 2.50–25.28] vs. 5.45 [range, 1.22–15.06]; *P* = 0.010), LN metastases (median SUV_max_, 8.86 [range, 3.51–23.33] vs. 3.84 [range, 1.01–9.75]; *P* = 0.002), and other metastases (median SUV_max_, 6.39 [range, 0.55–12.78] vs. 1.88 [range, 0.73–8.35]; *P* = 0.046) (Fig. 2A; Table 2). The number of metastatic lesions detected on [^18^F]FAPI-74 PET was significantly higher than that on [^18^F]FDG PET (Fig. 2B). In 6 patients, [^18^F]FAPI-74 PET detected more metastatic lesions than [^18^F]FDG PET, especially LN and peritoneal metastases in pancreatic cancer and LN metastases in breast cancer (Figs. 3 and 4). [^18^F]FAPI-74 PET showed clearer uptake in patients with lung adenocarcinoma (*n* = 2), even when [^18^F]FDG PET showed only faint uptake (Fig. 5).

**TABLE 2.** Head-to-Head Comparison of SUV_max_ Between [^18^F]FAPI-74 PET and [^18^F]FDG PET

		[^18^F]FAPI-74 PET					[^18^F]FDG PET				
Cancer type	State	Primary lesion	LN metastasis (1)	LN metastasis (2)	Other metastases	Location	Primary lesion	LN metastasis (1)	LN metastasis (2)	Other metastases	Location
Breast cancer	Before surgery	25.3	10.1				6.9	2.6			
Breast cancer	Before treatment	22.1	6.3	5.8*			7.3	1.7	ND (1.0)		
Breast cancer (case shown in Fig. 4)	Before treatment	19.6	23.3	11.9*			13.0	8.5	7.4		
Breast cancer	Recurrence		11.0	10.9*	12.8, 12.5	Liver		3.8	2.5	8.0, 8.4	Liver
Esophageal cancer	Before treatment	10.0					7.2				
Gastric cancer	Before treatment	5.0					2.2				
Gastric cancer	After NAC	8.2					5.5				
Gastric cancer	After NAC	ND	5.8				ND	9.8			
Gastric cancer	After NAC	3.4					3.1				
Gastric cancer	After NAC	7.2					10.4				
Lung cancer	Before surgery	15.4					13.3				
Lung cancer	Before surgery	6.4					4.7				
Lung cancer	Before surgery	5.1					2.5				
Lung cancer (case shown in Fig. 5)	Before surgery	2.5					1.2				
Lung cancer	Before surgery	11.8	3.5				14.9	4.9			
Head and neck	Before treatment	14.8	11.5	8.9*			15.1	7	4.4		
Pancreatic cancer	Before treatment	13.2					3.1				
Pancreatic cancer (case shown in Fig. 3)	Before treatment	9.4	5.4	6.5*	7.5, 5.3	Peritoneum	3.2	ND (2.3)	ND (1.5)	ND (2.0, 1.8)	Peritoneum
Pancreatic cancer	Recurrence	9.4	10.6	5.0*	4.6	Peritoneum	4.5	5.5	1.8	ND (1.2)	Peritoneum
Thymoma (type B3)	Recurrence				ND (0.6)	Pleura				ND (0.7)	Pleura

* Number of detected LN metastases is more than 3, and 2 major sites are shown here.

ND = not detected; NAC = neoadjuvant chemotherapy.

## DISCUSSION

In this study, we evaluated patients with various histopathologically confirmed cancers and benign lesions using [^18^F]FAPI-74 PET as an initial evaluation for a prospective clinical trial. [^18^F]FAPI-74 PET showed higher uptake in cancerous lesions than in nonmalignant lesions. [^18^F]FAPI-74 PET also showed significantly higher uptake in primary and metastatic lesions than did [^18^F]FDG PET. Reporting the superiority of [^18^F]FAPI-74 PET as an initial evaluation would aid the research community since the number of reports on [^18^F]FAPI-74 remains very limited.

Previous studies reported higher uptake of FAPI PET than of [^18^F]FDG in many types of cancers, including gastric, pancreatic, ovarian, and head and neck (7–10). It has been reported that FAPI PET detected a higher number of metastatic lesions than [^18^F]FDG PET did in patients with various types of cancers presenting with inconclusive [^18^F]FDG PET findings (7). The number of positive lesions was significantly higher in patients with gastric, lung, liver, and nasopharyngeal cancers. FAPI PET showed excellent sensitivity for peritoneal carcinomatosis of gastric, pancreatic, and ovarian cancers (8–10). We also observed peritoneal carcinomatosis in patients with pancreatic cancer, which could not be detected on [^18^F]FDG PET (Fig. 3). FAPI PET, including [^18^F]FAPI-74 PET, will contribute to accurate clinical staging and lead to proper patient management.

Two studies compared the uptake between FAPI PET and [^18^F]FDG PET in patients with histopathologically confirmed non–small cell lung cancer (11*,*^12^). Both studies reported that there was no statistically significant difference in the primary lesions in terms of SUV_max_, T/N ratio, or lesion detection. However, FAPI PET was significantly superior to [^18^F]FDG PET in the detection of LN, pleural, and bone metastases. In our cohort, we enrolled mainly preoperative patients without metastatic lesions. Although primary lesion detectability was similar in larger tumors, consistent with previous reports, [^18^F]FAPI-74 PET showed higher uptake in the ground-glass opacity lesions than did [^18^F]FDG PET, as shown in Figure 5.

FAPI PET is reported to be superior to [^18^F]FDG PET in detecting primary tumors, with high sensitivity in patients with breast cancer, as well as in detecting LN, hepatic, bone, and cerebral metastases because of its lower background activity (13–16). In our cohort, [^18^F]FAPI-74 PET showed a higher uptake than [^18^F]FDG, as shown in Figure 4. Additional LN metastasis was detected on [^18^F]FAPI-74 PET with histologic confirmation, which was not detected on [^18^F]FDG PET.

FAPI PET images must be interpreted carefully in terms of specificity. There are several pitfalls of noncancer uptake in benign lesions. A previous study reported that benign uptake occurs in bone degeneration, wound healing, the endometrium, and inflammation, including pancreatitis and pneumonia (17*,*^18^). Benign tumors sometimes show high FAPI uptake in renal angiomyolipomas, thyroid adenomas, necrotizing granulomas, and splenic hemangioma (18). Therefore, it is necessary to carefully interpret the possibility of benign or inflammatory uptake on [^18^F]FAPI-74 PET.

In this study, we observed a high uptake in postoperative changes or scars, granulomas, and radiation pneumonitis. One case study reported that FAPI PET showed high uptake in tuberculous granuloma (19). Although there have been no systematic reports of FAP expression in granulomas, granulomatous lesions may show high FAP expression. Regarding radiation pneumonitis, Qin et al. reported increased FAP expression and FAPI uptake in a rat model of radiation-induced lung damage (20). Further, Röhrich et al. reported increased uptake in fibrotic lung disease (21). In our study, there were no residual tumors among the high-uptake lesions on FAPI PET in a patient with lung cancer after chemoradiation therapy. It has been suggested that FAP expression is associated with radiation-induced fibrotic changes. Therefore, caution is required when using [^18^F]FAPI-74 PET to assess the therapeutic efficacy in lung cancer after radiation therapy.

Compared with [^68^Ga]-labeled FAPI PET probes, [^18^F]FAPI-74 might have the advantage of the shorter positron range of ^18^F (mean range in water, 0.6 mm) than of ^68^Ga (3.5 mm), thereby yielding a better image resolution, especially in small lesions (22). However, hepatobiliary excretion might affect the detectability of biliary tract cancer on [^18^F]FAPI-74 PET.

This study had some limitations. First, the number of patients was small, which should be finalized in the final report with a certain number of patients for each cancer type. Second, we performed [^18^F]FAPI-74 PET after [^18^F]FDG PET with a median interval of 18 d, and some [^18^F]FDG PET images were acquired using different PET scanners; thus, there may have been some resulting bias in the comparison. Third, we did not perform a comparison between immunohistochemical staining and FAP. Such a comparison should be included in future studies to confirm the specific uptake of [^18^F]FAPI-74 on PET and elucidate the FAP pathology in cancer-associated fibroblasts in greater detail.

## CONCLUSION

[^18^F]FAPI-74 PET showed higher uptake and detection rates in primary and metastatic lesions than did [^18^F]FDG PET. [^18^F]FAPI-74 PET is a promising novel diagnostic modality for various tumors, especially for precise staging before treatment, including characterization of tumor lesions before surgery. Moreover, ^18^F-labeled FAPI ligand might serve a higher demand in clinical care in the future.

## DISCLOSURE

This study was funded by the QiSS program of OPERA (grant JPMJOP1721) from the Japan Science and Technology Agency. Frederik Giesel is an advisor at ABX, Telix, SOFIE Biosciences, and α-Fusion and has shares in a consultancy group for iTheranostics. No other potential conflict of interest relevant to this article was reported.

## Notes

KEY POINTS **QUESTION:** Does [^18^F]FAPI-74 PET have higher uptake and detection rates in primary and metastatic lesions than [^18^F]FDG PET? **PERTINENT FINDINGS:** [^18^F]FAPI-74 PET showed significantly higher uptake in primary and metastatic lesions of various histopathologically confirmed cancers than did [^18^F]FDG PET. **IMPLICATIONS FOR PATIENT CARE:** The ^18^F-labeled FAPI ligand [^18^F]FAPI-74 PET is a promising novel diagnostic probe for various tumors that can serve a future higher demand with large-scale production.
